# Supplementary material for: Differential Responses of Colorectal Cancer Cell Lines to Enterococcus faecalis’ Strains Isolated from Healthy Donors and Colorectal Cancer Patients
Source: J Clin Med. 2019 Mar 20;8(3):388. doi: 10.3390/jcm8030388 (PMC6463247; doi:10.3390/jcm8030388)
Supplement: Supplementary file 1 [file jcm-08-00388-s001.pdf]

Supplementary material

1. *Genotypic characterization of E. faecalis' strains*

Table S1: Genotypic characterization of the virulence factors of the different *E. faecalis* strains

Virulencefactor	Identity	Query/HSP	Proteinfunction	Accession no.
cOB1	99,63	819/819		295112306
camE	99,6	501/501	sexpheromone cAM373 precursor	AF435438.1
gelE	100	1530/1530		355320029
fsrB	99,59	729/729	biofilmformation	JN246675.1
efaAfs	99,78	927/927		FP929058.1
tpx	100	510/510		CP002621.1
hylB	99,54	3015/3015		AE016830.1
SrtA	100	735/735		CP002491.1
hylA	98,64	4119/4119		AE016830.1
cad	100	930/930		CP002621.1
cCF10	100	828/828		CP002621.1
ElrA	99,95	2172/2172		CP002491.1
ace	99,52	1884/1884	collagenadhesin precursor	AF260876.1
ebpC	100	1884/1884		CP002491.1
ebpB	100	1431/1431		CP002491.1
ebpA	100	3312/3312		CP002491.1
gelE	99,87	1531/1530		CP002491.1
ElrA	99,68	2172/2172		CP002621.1
hylA	99,51	3266/3264		CP002491.1
SrtA	99,46	735/735		CP002491.1
camE	99,4	501/501	sexpheromone cAM373 precursor	AF435439.1
cad	100	930/930		CP002621.1
ebpA	99,34	3312/3312		CP002621.1
ebpB	99,3	1431/1431		AE016830.1
ebpC	99,36	1884/1884		295112306
ace	97,19	1743/1743	collagenadhesin precursor	AF260879.1
tpx	99,61	510/510		295112306
hylB	99,3	3015/3015		AE016830.1
cCF10	99,88	828/828		CP002621.1
efaAfs	100	927/927		FP929058.1
cOB1	99,63	819/819		CP002621.1
efaAfs	99,68	927/927		AE016830.1
cOB1	99,76	819/819		CP002621.1
ElrA	99,82	2172/2172		CP003726.1
agg	100	3906/3906	aggregationsubstance	AF454824.1
**cylL**	**100**	**207/207**		**AY032999.1**
**cylM**	**100**	**2982/2982**		**AE016830.1**
**cylB**	**99,81**	**2145/2145**		**AY032999.1**
camE	99,8	501/501	sexpheromone cAM373 precursor	AF435439.1
ace	96,71	1884/1884	collagenadhesin precursor	AF260877.1
ebpC	99,58	1884/1884		CP002491.1
ebpB	99,51	1431/1431		CP003726.1
ebpA	99,7	3312/3312		295112306
SrtA	99,32	735/735		AE016830.1
hylA	99,39	3266/3264		CP002491.1
cad	99,35	930/930		AE016830.1
cCF10	99,28	828/828		CP002621.1
tpx	100	510/510		CP003726.1
**espfm**	**92,01**	**3555/4938**		**CP003351.1**
gelE	100	1530/1530		CP003726.1
fsrB	100	729/729	biofilmformation	CP003726.1

camE	99,8	501/501	sexpheromone cAM373 precursor	AF435441.1
cCF10	99,76	828/828		CP002621.1
tpx	100	510/510		CP003726.1
hylB	99,6	3015/3015		CP002621.1
efaAfs	100	927/927		CP003726.1
cOB1	100	819/819		CP002491.1
ebpA	99,97	3312/3312		CP003726.1
ebpB	100	1431/1431		CP003726.1
ebpC	100	1884/1884		CP002491.1
ace	96,99	2025/2025	collagenadhesin precursor	AE016830.1
ElrA	100	2172/2172		CP003726.1
cad	99,78	930/930		CP002621.1
SrtA	100	735/735		CP003726.1
hylA	98,54	4119/4119		AE016830.1
gelE	99,41	1528/1530		CP003726.1
fsrB	100	729/729	biofilmformation	CP002621.1
efaAfs	99,89	927/927		CP002491.1
camE	100	501/501	sexpheromone cAM373 precursor	AF435441.1
cOB1	99,63	819/819		CP002621.1
SrtA	99,18	735/735		CP002491.1
hylA	98,64	4119/4119		AE016830.1
cad	100	930/930		CP003726.1
cCF10	99,88	828/828		CP002621.1
ace	97,24	1884/1884	collagenadhesin precursor	AF260877.1
ebpC	100	1884/1884		CP002491.1
ebpB	100	1431/1431		CP003726.1
ebpA	100	3312/3312		CP003726.1
hylB	99,57	3015/3015		CP002621.1
ElrA	99,45	2172/2172		CP003726.1
tpx	100	510/510		CP003726.1
cOB1	99,63	819/819		295112306
camE	99,6	501/501	sexpheromone cAM373 precursor	AF435438.1
gelE	100	1530/1530		355320029
fsrB	99,59	729/729	biofilmformation	JN246675.1
efaAfs	99,78	927/927		FP929058.1
tpx	100	510/510		CP002621.1
hylB	99,54	3015/3015		AE016830.1
SrtA	100	735/735		CP002491.1
hylA	98,64	4119/4119		AE016830.1
cad	100	930/930		CP002621.1
cCF10	100	828/828		CP002621.1
ElrA	99,95	2172/2172		CP002491.1
ace	96,99	2025/2025	collagenadhesin precursor	AF260874.1
ebpC	100	1884/1884		CP002491.1
ebpB	100	1431/1431		CP002491.1
ebpA	100	3312/3312		CP002491.1
camE	99,4	501/501	sexpheromone cAM373 precursor	AF435438.1
ebpA	99,49	3312/3312		CP003726.1
ebpB	99,65	1431/1431		CP003726.1
ebpC	99,26	1884/1884		295112306
ace	97,35	1884/1884	collagenadhesin precursor	AF260876.1
gelE	99,93	1530/1530		CP002621.1
fsrB	99,86	729/729	biofilmformation	CP002621.1
tpx	99,61	510/510		AE016830.1
cOB1	98,53	819/819		AE016830.1
efaAfs	99,78	927/927		FP929058.1
ElrA	98,71	2172/2172		CP003726.1
hylB	99,54	3015/3015		CP002621.1
SrtA	99,05	735/735		CP002491.1
hylA	98,93	3266/3264		CP002491.1
cCF10	99,76	828/828		CP002621.1

cad	99,89	930/930		CP002621.1
camE	100	501/501	sexpheromone cAM373 precursor	AF435438.1
gelE	99,87	1530/1530		CP002491.1
hylA	98,66	4119/4119		AE016830.1
tpx	99,8	510/510		CP003726.1
SrtA	99,46	735/735		CP002491.1
cad	99,35	930/930		AE016830.1
cOB1	99,63	819/819		CP002621.1
agg	95,69	3692/3906	aggregationsubstance	AF454824.1
ElrA	99,59	2172/2172		CP003726.1
efaAfs	100	927/927		FP929058.1
cCF10	100	828/828		FP929058.1
ebpA	99,37	3312/3312		CP003726.1
ebpB	99,65	1431/1431		CP002491.1
ebpC	99,68	1884/1884		CP002491.1
ace	100	1743/1743	collagenadhesin precursor	AF260879.1

2. *GC-MS analysis of SCFAs*

The MS acquisition was carried out in single ion monitoring (SIM) recording the characteristic ions of monitored SCFAs (Table 2).The quantitative SCFAs' evaluation was carried out by ratios between the area abundances of the analyses with the area abundances of respective labeled internal standards (isotopic dilution method). The ionic signals and the reference internal standard, used for the quantitation of each SCFA,were reported in Table 2.3 mL of pre-fermented medium sample was added of 50µL of ISTDs mixture, 1 mL of tert-butyl methyl ether and 50 µL of 1 M HCl solution in 15 mL centrifuge tube. Then, each tube was shaken in vortex apparatus for 2 minutes, centrifuged at 10000 rpm for 5 minutes, and finally the solvent layer was transferred in auto sampler vial and analyzed by GC-MS method. Each sample has been prepared and processed, by the previously described method , three times.

Table s2: Ionic signal used for quali/quantitation and relative ISTD of each SCFA acquired by the GC-MS method.

SCFAs	Quan. Ion	Qual. ion	ISTD
**Acetic**	60	-	[2H$_3$]Acetic
**Propionic**	74	73	[2H$_3$]Propionic
**iso-Butyric**	73	88	[2H$_7$]iso-Butyric
**Butyric**	60	73	[2H$_3$]Propionic
**iso-Valeric**	60	87	[2H$_9$]iso-Valeric
**Valeric**	60	87	[2H$_9$]iso-Valeric

The cell cycle phase distribution (propidium iodide staining) has beenestimated by flow cytometry. For that, we evaluated the effects of 48 hours exposure of bacterial metabolites on CLR1790 cells (considered as no tumor cell controls), and on HCT116 cells, which were the more responsive cells to the bacterial metabolites among the others. The results did not shown any accumulation in GO/G1, S or G2/M phases induced by bacterial metabolites (Figure 1).However, in according to the previous MTT assay (see the manuscript), we noted a trend to decreasing G0/G1 and increasing S phases, mainly with the metabolites of the EFH03 strain, which goes. Of note, we have documented that EFH03 was the most effectiveness strain on decreasing the cancer cell proliferation.

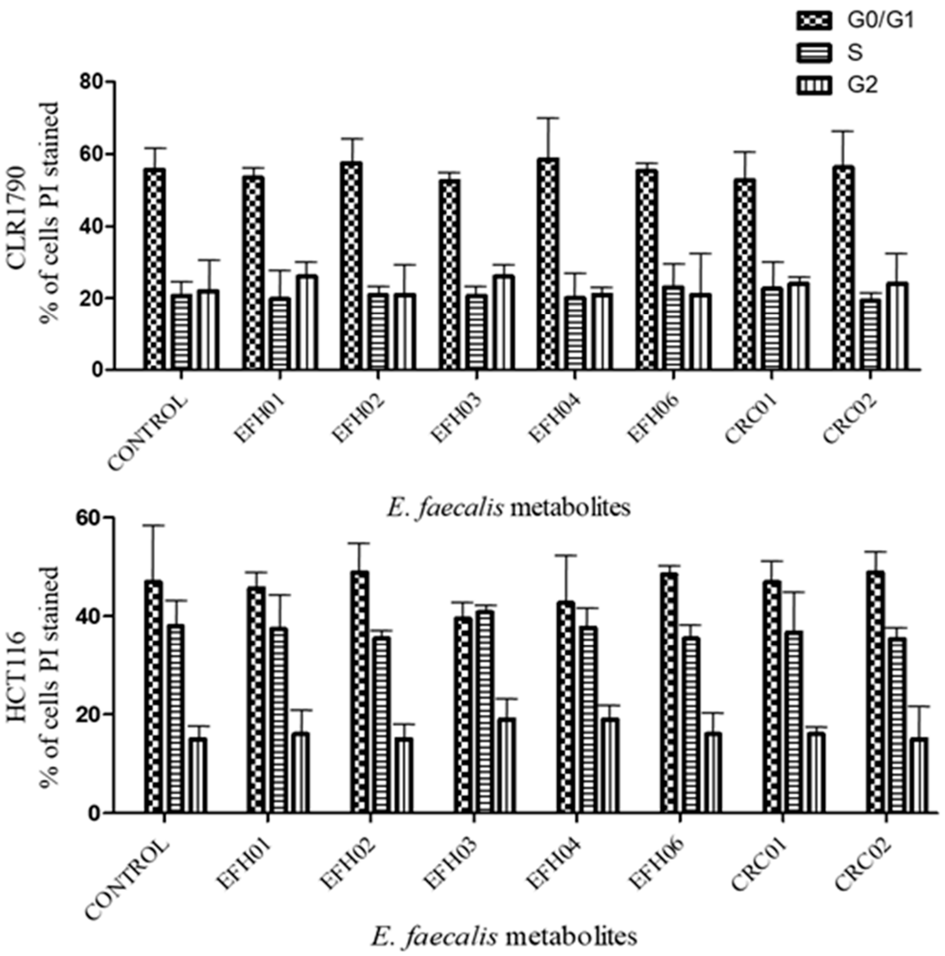

Figure s1: Cell cycle phase distribution of CLR1790 and HCT-116 cell lines after 48 hours of bacterial metabolites exposure. In total, 150 000 cells/well were seeded in multiwell dishes and exposed to bacterial metabolites. After medium removal, 400µl of solution containing 50 µg/mL propidium iodide, 0.1% w/v trisodium citrate and 0.1% NP40 was added. Samples were then incubated for 30 min at 4°C in the dark and nuclei analyzed with a FACS Canto flow cytometer (Becton Dickinson, Franklin Lakes, New Jersey, USA). All the experiments have been made in triplicate. Data are shown as mean +- sd.
